# Probiotic *Lactobacillus* Strains Stimulate the Inflammatory Response and Activate Human Macrophages

**DOI:** 10.1155/2017/4607491

**Published:** 2017-07-05

**Authors:** L. M. Rocha-Ramírez, R. A. Pérez-Solano, S. L. Castañón-Alonso, S. S. Moreno Guerrero, A. Ramírez Pacheco, M. García Garibay, C. Eslava

**Affiliations:** ^1^Departamento de Infectología, Hospital Infantil de México Federico Gómez, Dr. Márquez No. 162, Col Doctores, 06720 Delegación Cuauhtémoc, MEX, Mexico; ^2^Departamento de Inmunoquímica y Biología Celular, Hospital Infantil de México Federico Gómez, Dr. Márquez No. 162, Col Doctores, 06720 Delegación Cuauhtémoc, MEX, Mexico; ^3^Departamento de Hemato-Oncología, Hospital Infantil de México Federico Gómez, Dr. Márquez No. 162, Col Doctores, 06720 Delegación Cuauhtémoc, MEX, Mexico; ^4^Departamento de Biotecnología, Unidad Iztapalapa and Departamento de Ciencias de la Alimentación, Unidad Lerma, Universidad Autónoma Metropolitana, Av. San Rafael Atlixco No. 186, Col Vicentina, 09340 Mexico City, Mexico; ^5^Departamento de Salud Pública/División de Investigación, Facultad de Medicina, UNAM and Laboratorio de Patogenicidad Bacteriana, Unidad de Hemato-Oncología e Investigación, Hospital Infantil de México Federico Gómez, Dr. Márquez No. 162, Col Doctores, 06720 Delegación Cuauhtémoc, MEX, Mexico

## Abstract

*Lactobacilli* have been shown to promote health functions. In this study, we analyzed the mechanism by which four different strains of probiotics affected innate immunity, such as regulation of ROS, cytokines, phagocytosis, bactericidal activity, signaling by NF-*κ*B pp65, and TLR2 activation. The production of ROS was dependent on the concentration and species of *Lactobacillus*. The results obtained from the tested strains (*Lactobacillus rhamnosus* GG, *L. rhamnosus* KLSD, *L. helveticus* IMAU70129, and *L. casei* IMAU60214) showed that strains induced early proinflammatory cytokines such as IL-8,TNF-*α*, IL-12p70, and IL-6. However, IL-1*β* expression was induced only by *L. helveticus* and *L. casei* strains (after 24 h stimulation). Phagocytosis and bactericidal activity of macrophages against various pathogens, such as *S. aureus*, *S. typhimurium*, and *E. coli*, were increased by pretreatment with *Lactobacillus*. The nuclear translocation NF-*κ*B pp65 and TLR2-dependent signaling were also increased by treatment with the probiotics. Taken together, the experiments demonstrate that probiotic strains of *Lactobacillus* exert early immunostimulatory effects that may be directly linked to the initial inflammation of the response of human macrophages.

## 1. Introduction

The FAO and WHO (Food and Agriculture Organization of the United Nations and World Health Organization) define probiotics as “live microorganisms which when administered in adequate amounts confer a health benefit on the host” [1]. In particular, *Lactobacillus* is an important member of the probiotic bacteria that plays essential roles of immunomodulation in the intestinal mucosa [2]. Clinical and experimental studies of probiotic *Lactobacillus strains* have reported that these bacteria efficiently prevent and treat antibiotic-associated diarrhea, traveler's diarrhea, and infections caused by intestinal pathogens [3, 4]. In addition, some studies have shown that they provide a positive effect by promoting the secretion of immunoglobulin IgA and the production of antimicrobial molecules (i.e., bacteriocins), which are capable of inhibiting some intestinal pathogens [5]. In this context, the immunomodulatory effects of probiotic *Lactobacillus* strains have also been shown to decrease the inflammatory response under some pathological conditions such as necrotizing enterocolitis and allergies in children [6, 7]. In other in vitro studies, some strains produced an increase in the production of proinflammatory cytokines such as TNF-*α*, IL-12, and IL-8 whereas others produced increases in the secretion of anti-inflammatory cytokines such as IL-10 [8, 9]. A study by Lopez et al. [10] showed that UV-inactivated and live *Lactobacillus rhamnosus* GG (LGG) are equally effective in decreasing IL-8 in the intestinal epithelium. In addition, Li et al. [11] showed that live and heat-killed LGG are able to exert similar effects on the secretion of pro- and anti-inflammatory cytokines and chemokines when included in the diet of infant rats. However, these beneficial effects have been associated with only a limited number of strains, and other strains and species cannot be presumed to exert the same effects [12, 13]. The immune effects of probiotic bacteria have therefore been shown to be extremely diverse and strain-dependent in addition to cell type-specific.

Monocytes and macrophages are cellular components of the innate immune system that prevent the invasion of pathogens by releasing cytotoxic molecules such as reactive oxygen species (ROS) and by secreting proinflammatory cytokines such as TNF-*α* and IL-8 [14, 15]. Macrophages sense bacteria because bacteria express conserved pattern recognition receptors (PRRs), such as Toll-like receptors (TLRs). Macrophages also mediate responses when they recognize microbe-associated molecular patterns (MAMPs), which are expressed on the cell surface of probiotic bacteria [16]. Some studies have shown that TLR2 and TLR4 are constitutively expressed on macrophages [17]. The activation of TLR results in the induction of a signaling cascade that modulates the expression of various response genes such as cytokines and may activate signaling pathways, such as the NF-*k*B signaling pathway [18]. Miettinen et al. [19] showed that both viable and dead *L. rhamnosus* GG increased macrophage functions by activating NF-*k*B, STAT1, and STAT3 DNA-binding activity. Although immunological activities have been attributed to some probiotics, the molecular mechanisms underlying these effects have not been established. Determining the probiotic effects of *Lactobacillus* on the innate immune response and host health could support applications aimed at preventing and treating different diseases. A better understanding of how probiotic bacteria interact with host cells is therefore needed to optimize such applications.

## 2. Materials and Methods

### 2.1. Bacteria Strains and Growth Conditions

Four strains of lactic acid bacteria were used in this study: *Lactobacillus rhamnosus* GG, *Lactobacillus rhamnosus* KLDS*, Lactobacillus helveticus* IMAU70129, and *Lactobacillus casei* IMAU60214 previously isolated from commercial products by Cruz-Guerrero et al. [20, 21]. In the stimulation experiments, the *Lactobacillus* was cultured prior to use in MRS broth (Difco) overnight at 37°C. The bacterial cells were harvested at stationary phase using centrifugation (4000 ×g, 10 min) and washed twice with sterile physiological saline solution. The concentrated bacterial cells were inactivated by heating the cells to 85°C for 15 min. Frozen stocks of bacterial cells were stored in SSF at −80°C until use. Additionally, *Staphylococcus aureus* (ATCC 2913), *Escherichia coli* (ATCC 35218), and *Salmonella* enteric serovar *Typhimurium* (ATCC 14028) were grown overnight in tryptic soy broth (Difco Laboratories) at 37°C. After the cells were centrifuged (4000 ×g 5 min) and washed three times in sterile physiological saline, the bacterial strains were normalized to a density of ~10^8^ CFU/ml. In some experiments, the bacteria were inactivated using heat and the loss viability was confirmed by plating the inactivated bacteria on agar plates (at 37°C for 48 h). FITC-labeled bacteria (*S. aureus*, *S. typhimurim*, and *E. coli*) were killed by heating the cells at 80°C for 60 min and then resuspended at a concentration of 5 mg/ml in 50 mM carbonate buffer (pH 9.6). FITC isomer I (Sigma) was then added to the cells, which were incubated at 37°C for 30 min, and then washed with sterile PBS and stored at −80°C until use.

### 2.2. Macrophages Derived from Human Monocytes

Leukocyte-rich buffy coat was obtained from volunteer donors (Blood Bank, Children's Hospital of Mexico Federico Gomez), with approval from the local Ethics Committee. Mononuclear cells were separated using gradient centrifugation, as previously described [22]. Briefly, the interface mononuclear cells were washed twice with sterile physiological saline solution and then collected in Falcon tubes. The cells were immediately counted in a Neubauer chamber and cell viability was evaluated by staining the cells with trypan blue. To isolate the monocytes, we performed magnetic labeling with negative selection using a Monocyte Isolation Kit II (Miltenyi Biotec), according to the manufacturer's instructions. Purified monocytes were then differentiated into macrophages by culturing the cells in RPMI-1640 medium supplemented with 10% FBS (Gibco, Invitrogen USA) for 7 days.

### 2.3. ROS Production

ROS secretion was quantitated using chemiluminescence in a luminol-amplified system in an LKB-1251 luminometer, which coverts photons in a photomultitiplier tube into an electric current in the presence of a peroxidase such as HRP, according to the method described in Rellstab et al. [23]. Briefly, the macrophages were adhered to round glass coverslips (9 mm) at concentration of 1 × 10^5^ cells per coverslip and then placed in cuvettes containing 1 ml of Hank's solution (luminol 0.8 × 10^−4^ M and HRP at 4 U). After the cuvettes were warmed at 37°C for 10 min, baseline chemiluminescence was recorded. Subsequently, different concentrations of heat-inactivated *Lactobacillus* were added to the reaction mixture (e.g., MOI, 1 : 10, 1 : 100, 1 : 250, and 1 : 500). Opsonized zymosan (ZAS) and unstimulated macrophages (Hank's solution) were used as the positive and negative controls, respectively.

### 2.4. Cytokines

Macrophages were cultured and unstimulated or stimulated with heat-inactivated lactobacillus at a MOI of 500 : 1 (bacteria : macrophage), and the supernatants were collected after 6 and 24 h. Cytokine levels (IL-8, TNF-α, IL-6, IL-12p70, IL-1*β*, and IL-10) were quantified using an ELISA detection kit (BD OptEIA Pharmingen, San Diego, CA, USA) according to the manufacturer's instructions. A standard curve was used to calculate the concentrations of the different cytokines in pg/ml.

### 2.5. Macrophage Phagocytosis Assay

For the phagocytosis assay, 1 × 10^4^ macrophages in RPMI-1640 were plated onto 12 mm glass coverslips (Corning) and placed in sterile 24-well tissue culture plates (Costar). The cells were allowed to adhere in an atmosphere containing 5% CO_2_. Opsonized zymosan was added to the macrophages at (a MOI of 10 : 1). Simultaneously, macrophages in separate wells were pretreated with lactobacillus at a MOI (of 500 : 1 for 1 h at 37°C) and subsequently challenged with zymosan and opsonized bacteria such as *Staphylococcus aureus*, *E. coli*, and FITC-labeled *Salmonella typhimurium* a MOI of 10 : 1. To terminate phagocytosis, the cells were vigorously washed with PBS (pH 7.4) and then fixed in 4% paraformaldehyde (pH 7.4). Subsequently, the nuclei of the cells were stained with DAPI (1 mg/ml). Glass coverslips were air-dried and mounted onto glass microscope slides using Vectashield (Vysis). The phagocytic bacteria were visualized using an epifluorescence Zeiss Axioskop 2 microscope equipped with a Zeiss Axiocam (Zeiss AG, Oberkochen, DE). The percentage of ingesting bacteria was determined by evaluating 100 cells per field across four separate experiments. The phagocytic index was calculated as the percentage of cells performing phagocytosis using the mean number of particles per cells.

### 2.6. Macrophage Bactericidal Activity

Bacterial uptake and survival was measured using a gentamicin protection assay [24]. Before the cells were infected, 1 × 10^4^ macrophages in RPMI-1640 supplemented with 10% SFB were placed in sterile 12-well tissue culture plates (Costar). The cells were allowed to adhere in an atmosphere containing 5% CO_2_. Simultaneously, macrophages in separate wells were pretreated with lactobacillus at a MOI (of 500 : 1 for 1 h at 37°C) and subsequently challenged with *Escherichia coli*, *Staphylococcus aureus*, and *Salmonella* serovar *Typhimurium* at a MOI of 1 : 10. The cells were then incubated for 60 min at 37°C in atmosphere containing 5% CO_2_. The end of this incubation period was considered time 0. Each well was then washed and treated with media containing 200 *μ*g/ml gentamicin for 30 min at 37°C in an atmosphere containing 5% CO_2_ to kill any extracellular bacteria. The media was then replaced with media containing 20 *μ*g/ml gentamicin for the duration of the experiments. At time point 1 (120 min), the wells were washed 4 times with 1 ml of PBS each time. They were then incubated for 10 min at room temperature in sterile water containing 1% Triton-X-100 to lyse the macrophages. Dilutions of the resulting lysates were plated on LB agar plates and the number of viable intracellular bacteria was counted as colony-forming units (CFU).

### 2.7. NF-*κ*B (p65) Detection Using Indirect Immunofluorescence and Transnuclear Activity Assays

Human macrophages were attached to round glass coverslips (9 mm) and then either unstimulated or stimulated using zymosan (10 *μ*g/ml) and probiotic heat-inactivated lactobacillus at a MOI of 1 : 500. NF-*k*B activity was measured in the macrophages using indirect immunofluorescence with a Cellomics NF-*k*B kit (Thermo Scientific) according to the manufacturer's protocols. Fluorescence emitted within 30 min of the addition of different stimuli was photographed using an epifluorescence Zeiss Axioskop 2 microscope equipped with a Zeiss Axiocam (Zeiss AG, Oberkochen, DE). The percentage of cells that were positive for pp65 was determined in 100 cells per field across four separated experiments.

### 2.8. Transient Expression of TLR2 in HEK293 Cells (293-hTLR2)

Human embryonic kidney (HEK293-hTLR2) cells were obtained from InvivoGen (SanDiego, CA) and grown in 24-well culture plates in DMEM supplemented with 10% FCS and 10 *μ*g/ml blasticidin (InvivoGen) at 37°C in a 5% CO_2_ atmosphere. Cultures at 70–80% confluence were challenged with the probiotic bacteria lactobacillus at a MOI of 1 : 500. The plates were then incubated at 37°C for 24 h in an atmosphere containing 5% CO_2_. The supernatants were collected, and TLR activation was analyzed using ELISA for the quantity production of IL-8, according to the manufacturer's protocols. The control, cultures were stimulated with the TLR2 agonist zymosan 10 *μ*g/ml or LTA (10 *μ*g/ml). Blocking assays were performed using 10 *μ*g/ml of anti-hTLR2-IgA. The levels of IL-8 were then determined as described above for cytokines.

### 2.9. Statistical Analysis

The results are reported as the mean ± SD, for each experiment. A total of four donors were included in the duplicate samples. Differences between the conditions assays were analyzed using one-way analysis of variance (ANOVA) in Graph Pad Prism 5 Software. A *p* value of <0.05 was considered statistically significant.

## 3. Results

### 3.1. ROS Production

**Figure 1:**
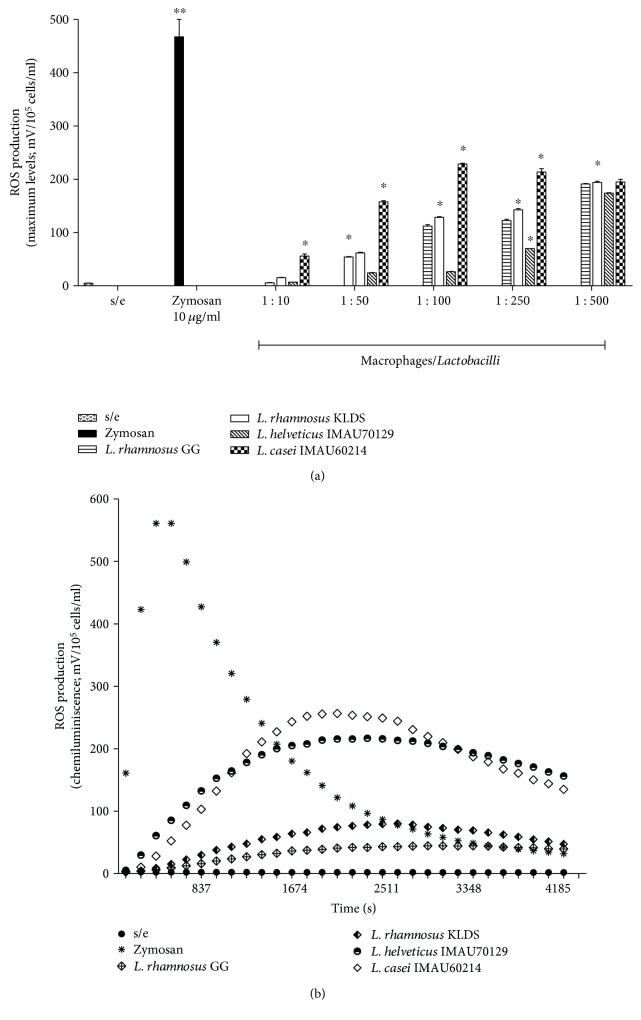
ROS production by human macrophages in response to probiotic heat-killed lactobacillus was measured by luminol-enhanced chemiluminescence. (a) Mixtures were prepared using 1 ml of Hank's solution, containing luminol 0.8 × 10^−4^ M, 4 U HRP, and lactobacillus bacteria at different MOI (1 : 1 and 1 : 500; bacteria : macrophages). The kinetics of the oxidative response induced in human macrophages by lactobacillus was monitored (b). The values shown represent four experiments that were performed in duplicate. Asterisks indicate the level of statistical significance between conditions. Significant difference: *p* < 0.05.

The effect of heat-killed lactobacillus on the generation of ROS species was measured using chemiluminescence (Figure 1). The results showed that adding heat-killed lactobacillus induced active respiratory bursts in human macrophages. ROS production was dependent on the bacteria: macrophage MOI and the species of *Lactobacillus*. The ROS-induced immunostimulatory effects were maximized at a MOI of 500 : 1 for *L. rhamnosus* GG, *L*. *rhamnosus* KLSD, *L. helveticus* IMAU70129, and *L*. *casei* IMAU60214, which produced peak chemiluminescence (190 ± 15, 196 ± 24, 175 ± 12, and 200 ± 14 Mv, resp.), (Figure 1(a)). However, the oxidative response induced by lactobacillus was lower than that induced by zymosan (200 ± 14 Mv versus 600 ± 20 Mv).

In addition, as shown in Figure 1(b), the zymosan-induced ROS production kinetic was fast and short (500 sec), whereas the heat-killed lactobacillus-induced ROS production kinetic was delayed (2000 sec) and took longer to return to baseline. These results indicate that heat-killed lactobacillus activates ROS production in human macrophages.

### 3.2. Cytokine Production in Macrophages

**Figure 2:**
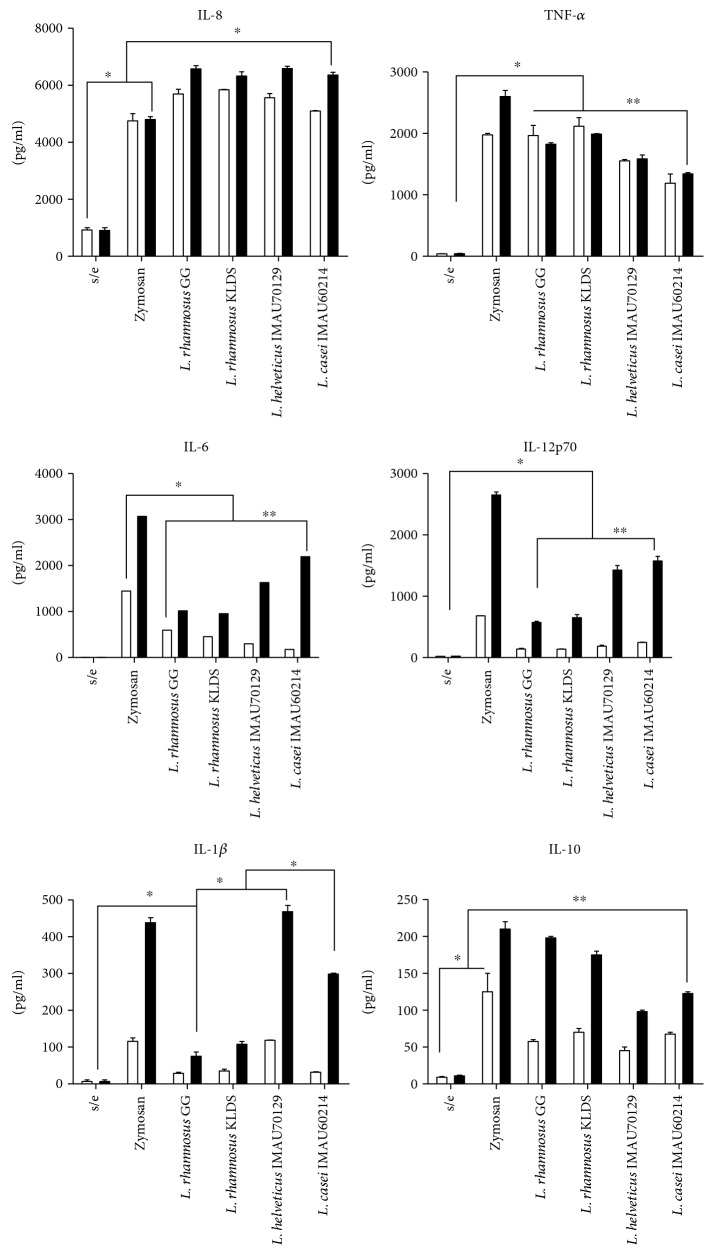
Cytokine production in human macrophages that were stimulated using heat-killed lactobacillus. The supernatants of stimulated macrophages were analyzed using ELISA to determine the level of expression of the cytokines IL-8, TNF-*α*, IL-6, IL-12, IL-1*β*, and IL-10. The macrophages were treated with zymosan (10 *μ*g/ml) or heat-killed strains of lactobacillus at a MOI of 1 : 500 or untreated (s/e). Cytokine concentrations were determined at 6 h (□) and 24 h (■) after stimulation. The results are shown as the means ± of the standard deviations and are representative of four independent experiments. Significance was set at *p* < 0.05 (∗) in the comparison between 6 and 24 h of stimulation. *p* > 0.05 (∗∗).

Macrophages treated with *Lactobacillus* were induced to produce cytokines between 6 and 24 h after the treatment began. In macrophages challenged with a MOI 1 : 500 of heat-inactivated lactobacillus, the maximum concentration of IL-8 was observed at 6 h (Figure 2). All strains of lactobacillus induced a significant level of IL-8. The results showed that in the treated macrophages, there was one logarithmic increase more than what was observed in the untreated macrophages, but similar values to those in the zymosan-stimulated macrophages (Figure 2). The level of IL-8 produced after 24 h was not different across the groups and remained high in the cells treated with all probiotic strains of lactobacillus. The level obtained of TNF-*α* was similar across treatments (Figure 2). In contrast, the maximum concentration of IL-6 was obtained after of 24 h and the level of this cytokine was different across the treatments (Figure 2). *L. casei* IMAU60214 and *L. helveticus* IMA70129 induced the highest levels of IL-6 (e.g., higher than levels produced by *L. rhamnosus* KLSD and *L. rhamnosus* GG). In addition, the synthesis of the cytokine IL-12p70 was significantly higher in these strains. IL-1*β* expression was increased after 24 only following stimulation with *L. helveticus* IMA70129 and *L. casei* IMAU60214. In contrast, neither *L. rhamnosus* GG nor *L. rhamnosus* KLSD induced a significant increase in the cytokine IL-1*β* (Figure 2). In this experiment, we found that all strains of probiotic lactobacillus induced the production of IL-10 later, after 24 h, and the levels produced were different across the strains (Figure 2). These data collectively indicate that each one of the strains of lactobacillus induced a strong inflammatory response in macrophages.

### 3.3. *Lactobacillus* Modulates Phagocytosis in Macrophages

**Figure 3:**
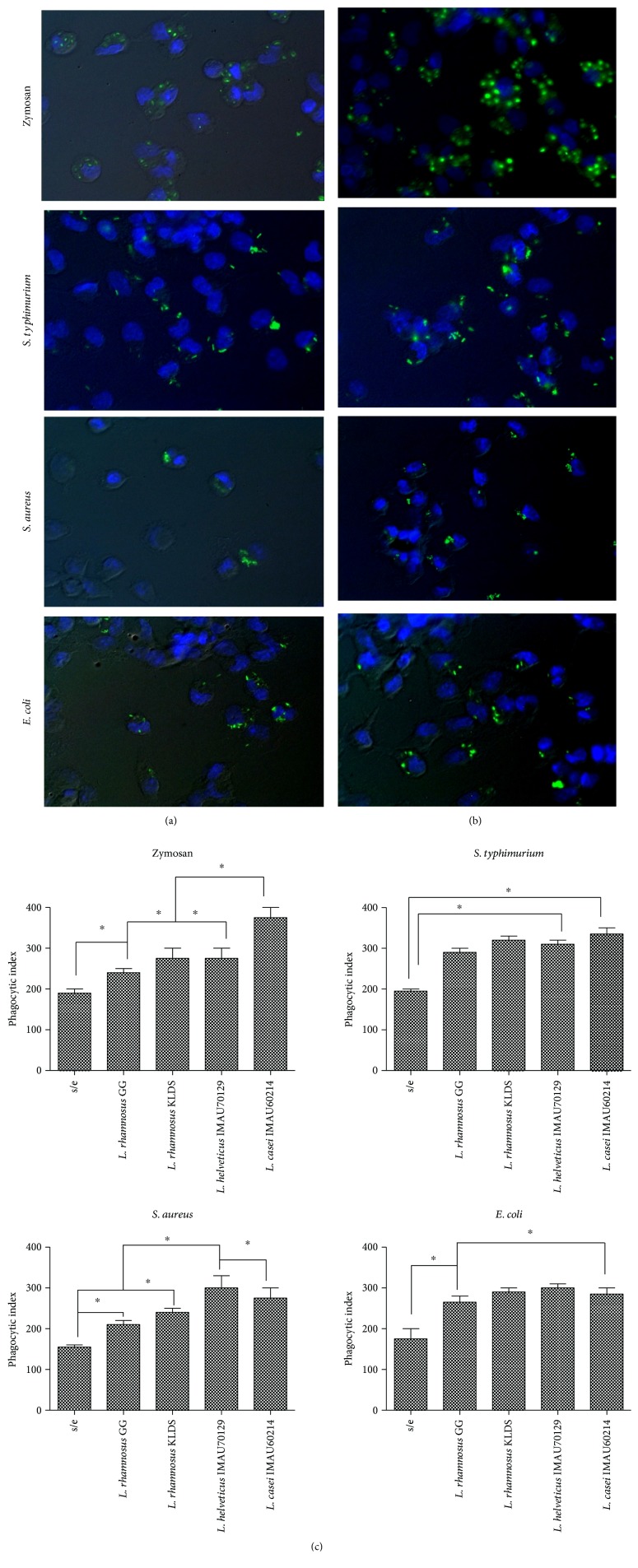
Effect of heat-killed lactobacillus on phagocytosis in human macrophages. Cells (1 × 10^4^) were treated with heat-killed strains of lactobacillus at a MOI of 1 : 500 for 1 h before zymosan particles, *S. aureus*, *S. typhimurium*, or *E. coli* (each at a MOI of 1 : 10) were added. Phagocytosis was allowed to proceed for 1 h at 37°C in an atmosphere containing 5% C0_2_, before phagocytosis was terminated. The cells were then fixed, and the nuclei were stained with DAPI. Macrophages, untreated (a) and treated (b) cells. Fluorescence photomicroscopy in macrophages treated with different stimuli was performed using an epifluorescence Zeiss Axioskop2 microscope equipped with a Zeiss Axiocam (Zeiss AG, Oberkochen, DE). This figure shows the representative results with the strain (*L. casei* IMAU60214). (c) Heat-killed lactobacillus enhanced the phagocytic index of human macrophages. The ingestion of cells was determined in macrophages that were incubated in medium alone or preincubated with lactobacillus strains. The following formula was used: the percentage of cells undergoing phagocytosis × the number of particles per cells. The cells were incubated with zymosan, *S. typhimurium*, *S. aureus*, and *E. coli*, as indicated. Asterisks indicate a significant difference between conditions (*p* > 0.05).

The phagocytosis of bacteria is an effective mechanism employed by the innate immune response. We therefore preincubated macrophages with probiotic strains of lactobacillus to determine their ability to increase phagocytosis in response to challenge with different pathogens (Figure 3). In this study, we applied four previously selected stimuli and zymosan (prepared from the yeast *Saccharomyces cerevisiae*) to two species of Gram-negative (*S. typhimurium* and *E. coli*) and one species of Gram-positive (*S. aureus*) microorganism. In cells challenged with FITC-labeled phagocytic stimuli, the increased rate of ingestion of phagocytized particles was higher in macrophages that were pretreated for 1 h than in untreated cell (Figure 3). In addition, pretreatment with lactobacillus resulted in significantly higher efficiency of macrophage phagocytosis than was observed in the untreated cells (Figure 3(b)). *Lactobacillus helveticus* IMAU70129 and *L. casei* IMAU60214 most strongly activated the phagocytic functions of the macrophages. However, in summary, all of lactobacillus strains exerted a positive effect on the ingestion of FITC-labeled bacteria, including *S. aureus*, *S. typhimurium*, *E. coli*, and yeast (zymosan) by human macrophages (Figure 3(c)).

### 3.4. Bactericidal Activity

**Figure 4:**
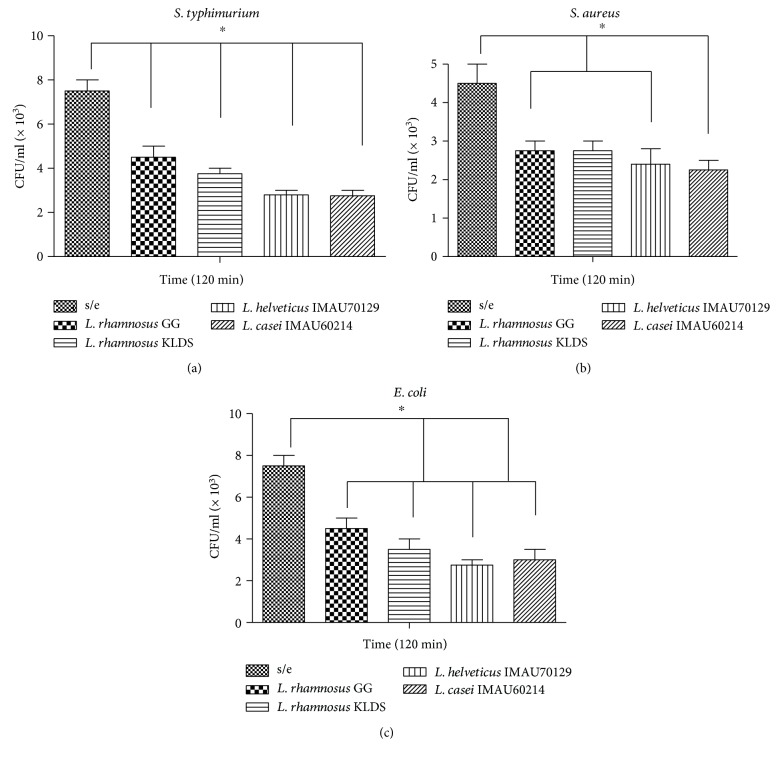
Effect of lactobacillus on the bacterial activity of macrophages against the pathogens *S. typhimurium, S. aureus*, and *E. coli*. Human macrophages were infected with (a) *S. typhimurium*, (b) *S. aureus*, or (c) *E. coli* and then incubated for 90 min at 37°C to allow the bacteria to be internalized. External bacteria were then killed by applying gentamicin for 30 min at 37°C. Samples were taken immediately before gentamicin treatment (0 h) and every 120 min thereafter to determine viable counts following Triton X-100 lysis. The results are expressed as CFU per milliliter with means ± SD from no fewer than four separate experiments that were performed in duplicate using macrophages obtained from different human donors. Asterisks indicate a significant difference between conditions (*p* < 0.05).

Macrophages were infected as described in the Materials and Methods. As shown in Figure 4, preincubating human macrophages for 120 min with heat-killed lactobacillus induced a decrease in the number of viable intracellular *S. typhimurium* (Figure 4(a)). The effects of this treatment on bactericidal activity were similar to those observed in cells treated with *E. coli* (Figure 4(b)) and *S. aureus* (Figure 4(c)). Furthermore, these results showed that was no difference in these effects across different species of *Lactobacillus*. Taken together, these data confirm that *Lactobacillus* bacteria restrict the survival of these pathogens.

### 3.5. *Lactobacillus* Bacteria Induce the Translocation of NF-*κ*B

**Figure 5:**
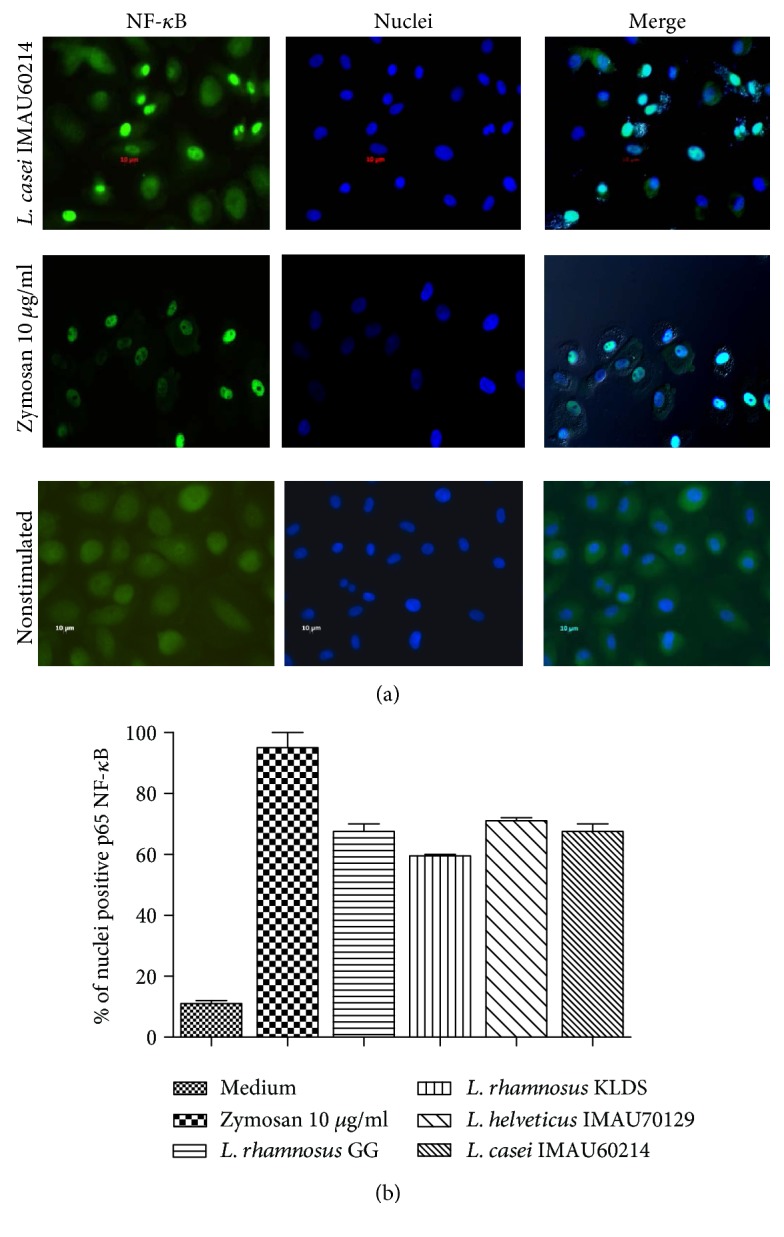
Immunofluorescence analysis of NF-*k*B activation. Human macrophages were stimulated for 30 min with heat-killed lactobacillus at a MOI of 1 : 500, zymosan (positive control) or left unstimulated. (a) Nuclear translocation was defined as blue fluorescence (Hoechst staining), and the NF-*k*B pp65 subunit was identified using green fluorescence (Dylight 488). (b) The percentage of nuclei that were positive for pp65. The results represent three independent experiments.

Various proinflammatory and anti-inflammatory cytokines are known to be regulated at least in part by the transcription activator NF-*k*B. As shown in Figure 5, the activation and translocation NF-*k*B pp65 were induced in macrophages challenged with lactobacillus (Figure 5(a)). The expression of the NF-*k*B translocation factor was observed in more than 60% of the treated cells after 30 min of incubation (Figure 5(b)). In macrophages stimulated with zymosan, more than 90% of the cells expressed this factor. However, in untreated macrophages, approximately 20% of the cells expressed it (Figure 5). Taken together, these indicate that heat-killed lactobacillus activates the expression of the NF-*k*B factor pp65.

### 3.6. TLR2 Mediates the Recognition of *Lactobacillus*

**Figure 6:**
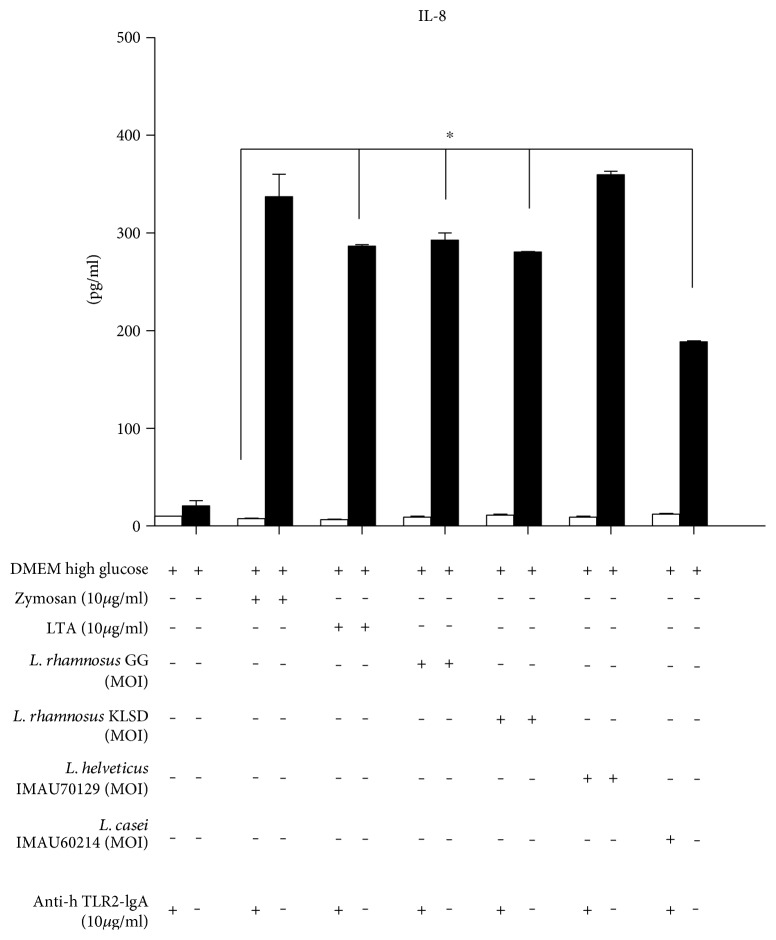
Transfection assay of HEK293-hTLR2 cells showing IL-8 was secreted in response to strains of lactobacillus. The recognition of lactobacillus strains was evaluated using a model in which HEK293-hTLR2 cells were transfected, and then blocking assays were performed using anti-hTLR2-IgA antibodies at a concentration of 10 *μ*g/ml. *p* < 0.05 (∗).

We next sought to determine whether TLR2 was stimulated by lactobacillus in HEK293 cells. The results showed that the concentration of IL-8 induced in response to challenge with heat-killed *Lactobacillus* was similar to that produced by an agonist of TLR2, zymosan, and LTA (Figure 6). In addition, to verify that *Lactobacillus* plays a role in TLR2 recognition in inflammatory responses in human macrophages, we performed a blocking assay by applying anti-hTLR2-IgA antibodies at a concentration of 10 *μ*g/ml for 30 min and then adding the stimuli. As shown in Figure 6, after 24 h, the production of IL-8 was nearly completely inhibited by all strains of *Lactobacillus*. This inhibitory effect on IL-8 production was also induced by the TLR2 agonist ligands zymosan and LTA. These results suggest that TLR2 participates in cytokine-mediated proinflammatory responses.

## 4. Discussion

In intestinal homeostasis, macrophages play an important role by acting as immunological sentinels in the gastrointestinal tract [25]. Most of these cells infiltrate the lamina propria, which produces a variety of molecules (cytokines) that regulate and activate the innate immune response. This infiltration of macrophages, which are derived from monocytes, into the intestine may initiate an inflammatory response, in response to infection, and disruptions in the homeostasis of these cells may contribute to the immunological damage observed in chronic inflammatory diseases [26]. Several groups have proposed that intestinal homeostasis can be restored by administering supplements containing fermented dairy products and beneficial microorganisms (probiotics), such as *Lactobacillus*. In this study, we evaluated the immunological impact of four probiotic bacteria in the genus *Lactobacillus* (i.e., *L. rhamnosus* GG, *L. rhamnosus* KLDS, *L. helveticus* IMAU70129, and *L. casei* IMAU60214) on the induction of the synthesis of inflammatory mediators, including ROS. The immunological role of ROS has been highlighted in individuals with defects in the biochemical machinery required for protein synthesis. ROS entities are extremely toxic and have deleterious effects on a variety of pathogens, such as fungi, parasites, and bacteria [27]. Activated mononuclear phagocytes (i.e., monocytes and macrophages) are the main source of microbicidal ROS. We demonstrated that in macrophages, the production of ROS in response to challenge with lactic bacteria was dependent on the concentration and species of bacteria. Both of the probiotic bacteria used in this study (*L. casei* IMAU60214 and *L. helveticus* IMAU70129) induced the highest levels of ROS in human macrophages obtained from healthy hosts. In support of these results, Marcinkiewicz et al. [28] demonstrated that ROS production was also induced in murine macrophage stimulated with lactobacillus. Likewise, the production of new ROS molecules in response to lactobacillus was associated with the mechanisms underlying IL-12 synthesis [29].

In contrast, other investigations have demonstrated that free extracts of LGG and *L. paracasei* had antioxidant activities in cells grown in the presence of superoxide dismutase [30]. The variability in the biological effects exerted by different probiotics may be associated with a number of factors, such as secreted factors, including bacteriocins, lactic acid, short chain fatty acids, and the presence of SOD activity. Different studies have reported that the probiotic effects of different lactobacillus species affect the immunomodulatory capacity of various cellular components in the innate and mucosal immune systems, such as T, B, and NK lymphocytes [31]. We demonstrated that the heat-killed lactobacillus *L. rhamnosus* GG, *L. rhamnosus* KLSD, *L. helveticus* IMAU70129, and *L*. *caseí* IMAU60214 induce the production of the cytokine IL-8 in human macrophages. This chemokine plays a very important role in the recruitment of others immune cells during an inflammatory response [32]. The chemokine IL-8 is produced during the early stages of the interaction lactobacillus and macrophages (i.e., within 6 h of stimulation), and this response was sustained for 24 h at much higher levels (> 2000 pg/ml) than that of the production of other cytokines analyzed in this study. In similar studies, other authors have demonstrated that the TH1 lymphocytes display chemotaxis in response to the expression of the IL-8 mRNA and other soluble factors derived from LGG [33]. In contrast, Jiang et al. [34] showed that IL-8 synthesis was not induced when lines of epithelial intestinal (i.e., CaCo2, T84, and HT-29) and THP-1 monocyte cells were stimulated with *L. reuteri*. These discrepancies were attributed to differences in the study models and the types of cells used in them. In the current study, we also demonstrated that TNF-*α* and IL-8 were similarly induced and that they were maintained at high levels for 24 h. Both TNF-*α* and IL-8 are proteins that play very important biological roles in the regulation of the inflammatory responses of macrophages [35]. These results collectively suggest that the probiotic strains analyzed in this study have immunostimulatory effects on the synthesis of high level cytokines, including IL-6, IL-12, and IL-1*β*, for at least 24 h in human macrophages. One interesting result of this study was that the production of IL-1*β*, which plays a very important role in the costimulation of T lymphocyte functions, was not induced in response to all strains. Part of the impact of lactobacillus on the regulation of the immune response was to induce the synthesis of IL-10, which was observed at high levels at 24 h after stimulation. IL-10 has also been shown to play a role in chronic gastrointestinal problems, and its modulation by probiotic bacteria has been observed in patients with ulcerative colitis and inflammatory bowel disease [36]. There is no doubt that IL-10 downregulates proinflammatory cascades. We also demonstrated that these lactic bacteria had an impact on the phagocytic activity of macrophages against extracellular pathogens such as *S. aureus* and *E. coli* and intracellular pathogens such as *S. typhimurium*. In support of these results, a previous study showed that in a murine model supplementing the diet with *L. plantarum* CGMC 1.557 for 20 days enhanced phagocytic activity in macrophages [37]. In addition, Kausahal et al. [38] demonstrated that *Lactobacillus acidophilus* and *Bifidobacterium bifidum* improved the phagocytic potential of macrophages in aged mice. However, the signaling induced by the production of several mediators, such as cytokines, involves the activation of downstream transcription factors, such as NF-*k*B [39]. In the present work, we demonstrated that NF-*k*B activated an inflammatory response in response to heat-inactivated lactobacillus. Similar reports have shown that stimulation with *L. casei* induced the MAPK and NF-*k*B signaling pathways, which was associated with the secretion of the cytokines TNF-*α* and IL-12 from murine spleen cells [40]. *L. rhamnosus* GG also initiated signaling cascades including the NF-*k*B and STAT signaling pathways in human macrophages. In this study, we also found that in combination with the activation of NF-*k*B, TLR2 also plays an important role by activating the synthesis of IL-8 in HEK-hTLR2 cells in response to stimulation with lactobacillus (*Lactobacillus rhamnosus* GG, *L. rhamnosus* KLSD, *L. helveticu*s IMAU70129, and *L. casei* IMAU60214). In contrast, Shida et al. [41] reported that PGN derived from *L. jonhsonii* and *L. plantarum* exerted inhibitory effects via both TLR2-dependent and independent mechanisms. In other studies, using murine macrophages, stimulation with LTA reversed the ability of some lactobacillus strains to induce the synthesis of IL-12 by increasing the production of IL-10 via a mechanism involving the activation of ERK family dependent effects on the activation of TLR2 [42]. Other investigations have indicated that viable and lyophilized lactobacillus exerts different effects on immunomodulation and that these differences are partially attributable to the involvement of the TLR receptor but not TLR4 and TLR9 [43].

Finally, investigations into the mechanisms underlying the activation of macrophages by lactobacillus probiotics are important, and the results of studies such as this one suggest that this group of lactic bacteria, including *Lactobacillus rhamnosus* GG, *L. rhamnosus* KLSD, *L. helveticu*s IMAU70129, and *L. casei* IMAU60214, has potential adjuvant effects on the immune response of host organisms.

## 5. Conclusion

Four heat-killed lactobacillus probiotic strains exerted immunostimulatory properties by activating the in vitro inflammatory response of macrophages via mechanism involving the synthesis of proinflammatory mediators, including cytokines, ROS, and participation in signaling cascades, such as the NF-*k*B and TLR2 pathways. These observations suggest that the properties of heat-killed probiotics may improve the innate immune response. However, in vitro tests not necessarily represent which may occur in vivo, so it is important to evaluate the effect of probiotics in models with immunosuppressed animals and clinical studies in humans. Depending on the results obtained, the effect of these probiotics as potential immunomodulators in immunocompromised hosts could be evaluated.

## Abbreviations

PRRs: Pattern recognition receptors
TLRs: Toll-like receptors
MAMPs: Microbe-associated molecular patterns.

## References

[1] Reid G., Food and Agricultural Organization of the United Nation and the WHO (2005). The importance of guidelines in the development and application of probiotics. *Current Pharmaceutical Design*.

[2] Delcenserie V., Martel D., Lamoureux M., Amiot J., Boutin Y., Roy D. (2008). Immunomodulatory effects of probiotics in the intestinal tract. *Current Issues in Molecular Biology*.

[3] Vanderhoof J. A., Mitmesser S. H. (2010). Probiotics in the management of children with allergy and other disorders of intestinal inflammation. *Beneficial Microbes*.

[4] Nixon A. F., Cunningham S. J., Cohen H. W., Crain E. F. (2012). The effect of lactobacillus GG on acute diarrheal illness in the pediatric emergency department. *Pediatric Emergency Care*.

[5] Gillor O., Etzion A., Riley M. A. (2008). The dual role of bacteriocins as anti- and probiotics. *Applied Microbiology and Biotechnology*.

[6] Robinson J1. (2014). Cochrane in context: probiotics for prevention of necrotizing enterocolitis in preterm infants. *Evidence-Based Child Health*.

[7] West C. E., Hammarström M. L., Hernell O. (2013). Probiotics in primary prevention of allergic disease—follow-up at 8-9 years of age. *Allergy*.

[8] Grimoud J., Durand H., de Souza S. (2010). In vitro screening of probiotics and synbiotics according to anti-inflammatory and anti-proliferative effects. *International Journal of Food Microbiology*.

[9] Jensen H., Drømtorp S. M., Axelsson L., Grimmer S. (2015). Immunomodulation of monocytes by probiotic and selected lactic acid bacteria. *Probiotics Antimicrobial Proteins*.

[10] Lopez M., Li N., Kataria J., Russell M., Neu J. (2008). Live and ultraviolet-inactivated Lactobacillus rhamnosus GG decrease flagellin-induced interleukin-8 production in Caco-2 cells. *The Journal of Nutrition*.

[11] Li N., Russell W. M., Douglas-escobar M., Hauser N., Lopez M., Neu J. (2009). Live and heat-killed Lactobacillus rhamnosus GG: effects on proinflammatory and anti-inflammatory cytokines/chemokines in gastrostomy-fed infant rats. *Pediatric Research*.

[12] Zhang Y., Zhang L., Du M. (2011). Antimicrobial activity against Shigellasonnei and probiotic properties of wild lactobacillus from fermented food. *Microbiological Research*.

[13] Habil N., Al-Murrani W., Beal J., Foey A. D. (2011). Probiotic bacterial strains differentially modulate macrophage cytokine production in a strain-dependent and cell subset-specific manner. *Beneficial Microbes*.

[14] Suzuki N., Mittler R. (2012). Reactive oxygen species-dependent wound responses in animals and plants. *Free Radical Biology and Medicine*.

[15] Franken L., Schiwon M., Kurts C. (2016). Macrophages: sentinels and regulators of the immune system. *Cellular Microbiology*.

[16] Lebeer S., Vanderleyden J., De Keersmaecker S. C. (2010). Host interactions of probiotic bacterial surface molecules: comparison with commensals and pathogens. *Nature Reviews. Microbiology*.

[17] O'Mahony D. S., Pham U., Iyer R., Hawn T. R., Liles W. C. (2008). Differential constitutive and cytokine-modulated expression of human Toll-like receptors in primary neutrophils, monocytes, and macrophages. *International Journal of Medicine Sciences*.

[18] Rahman M. M., McFadden G. (2011). Modulation of NF-κB signalling by microbial pathogens. *Nature Reviews. Microbiology*.

[19] Miettinen M., Lehtonen A., Julkunen I., Matikainen S. (2000). Lactobacilli and Streptococci activate NF-kappa B and STAT signaling pathways in human macrophages. *Journal of Immunology*.

[20] Cruz-Guerrero A., Hernández-Sánchez H., Rodríguez-Serrano G., Gómez-Ruiz L., García-Garibay M., Figueroa-González I. (2014). Commercial probiotic bacteria and prebiotic carbohydrates: a fundamental study on prebiotics uptake, antimicrobials production and inhibition of pathogens. *Journal of the Science of Food and Agriculture*.

[21] Escamilla-Lozano Y., García-Garibay M., López-Munguía-Canales A., Gómez-Ruiz L., Rodríguez-Serrano G., Cruz-Guerrero A. (2015). Synthesis of a-l-fucosidase in different strains of lactic acid bacteria. *Revista Mexicana de Ingeniería Química*.

[22] Rocha-Ramírez L. M., Hernández-Chiñas U., Baños-Rojas D. (2016). Pet serine protease from enteroaggregative *Escherichia coli* stimulates the inflammatory response activating human macrophages. *BMC Microbiology*.

[23] Rellstab P., Schaffner A. (1989). Endotoxin suppresses the generation of O2- and H2O2 by resting and lymphokine-activated human blood-derived macrophages. *Journal of Immunology*.

[24] Stevanin T. M., Poole R. K., Demoncheaux E. A., Read R. C. (2002). Flavohemoglobin Hmp protects Salmonella enterica serovar typhimurium from nitric oxide-related killing by human macrophages. *Infection and Immunity*.

[25] MacDonald T. T., Monteleone I., Fantini M. C., Monteleone G. (2011). Regulation of homeostasis and inflammation in the intestine. *Gastroenterology*.

[26] van Lierop P. P., Samsom J. N., Escher J. C., Nieuwenhuis E. E. (2009). Role of the innate immune system in the pathogenesis of inflammatory bowel disease. *Journal of Pediatric Gastroenterology and Nutrition*.

[27] Fang F. C. (2011). Antimicrobial actions of reactive oxygen species. *MBio*.

[28] Marcinkiewicz J., Ciszek M., Bobek M. (2007). Differential inflammatory mediator response in vitro from murine macrophages to lactobacilli and pathogenic intestinal bacteria. *International Journal of Experimental Pathology*.

[29] Ichikawa S., Miyake M., Fujii R., Konishi Y. (2012). MyD88 associated ROS generation is crucial for Lactobacillus induced IL-12 production in macrophage. *PLoS One*.

[30] Sun J., Hu X. L., Le G. W., Shi Y. H. (2010). Lactobacilli prevent hydroxy radical production and inhibit *Escherichia coli* and *Enterococcus* growth in system mimicking colon fermentation. *Letters in Applied Microbiology*.

[31] Forsythe P., Bienenstock J. (2010). Immunomodulation by commensal and probiotic bacteria. *Immunological Investigations*.

[32] Griffith J. W., Sokol C. L., Luster A. D. (2014). Chemokines and chemokine receptors positioning cells for host defense and immunity. *Annual Review of Immunology*.

[33] Veckman V., Miettinen M., Matikainen S. (2003). Lactobacilli and streptococci induce inflammatory chemokine production in human macrophages that stimulates Th1 cell chemotaxis. *Journal of Leukocyte Biology*.

[34] Jiang Y., Lü X., Man C. (2012). Lactobacillus acidophilus induces cytokine and chemokine production via NF-κB and p38 mitogen-activated protein kinase signaling pathways in intestinal epithelial cells. *Clinical and Vaccine Immunology*.

[35] Commins S. P., Borish L., Steinke J. W. (2010). Immunologic messenger molecules: cytokines, interferons, and chemokines. *Journal of Allergy and Clinical Immunology*.

[36] De Moreno de LeBlanc A., del Carmen S., Zurita-Turk M. (2011). Importance of IL-10 modulation by probiotic microorganisms in gastrointestinal inflammatory diseases. *ISR Gastroenterology*.

[37] Ren D., Li C., Qin Y. (2015). Evaluation of immunomodulatory activity of two potential probiotic Lactobacillus strains by in vivo tests. *Anaerobe*.

[38] Kaushal D., Kansal V. K. (2014). Dahi containing Lactobacillus acidophilus and Bifidobacterium bifidum improves phagocytic potential of macrophages in aged mice. *Journal of Food Science and Technology*.

[39] Napetschnig J., Wu H. (2013). Molecular basis of NF-κB signaling. *Annual Review of Biophysics*.

[40] Kim Y. G., Ohta T., Takahashi T. (2006). Probiotic Lactobacillus casei activates innate immunity via NF-kappa B and p38 MAP kinase signaling pathways. *Microbes and Infection*.

[41] Shida K., Kiyoshima-Shibata J., Kaji R., Nagaoka M., Nanno M. (2009). Peptidoglycan from lactobacilli inhibits interleukin-12 production by macrophages induced by Lactobacillus casei through Toll-like receptor 2-dependent and independent mechanisms. *Immunology*.

[42] Kaji R., Kiyoshima-Shibata J., Nagaoka M., Nanno M., Shida K. (2010). Bacterial teichoic acids reverse predominant IL-12 production induced by certain lactobacillus strains into predominant IL-10 production via TLR2-dependent ERK activation in macrophages. *Journal of Immunology*.

[43] Cai S., Bay B. H., Lee Y. K., Lu J., Mahendran R. (2010). Live and lyophilized Lactobacillus species elicit differential immunomodulatory effects on immune cells. *FEMS Microbiology Letters*.

